# Supplementary material for: Population dynamics of immunological synapse formation induced by bispecific T cell engagers predict clinical pharmacodynamics and treatment resistance
Source: eLife. 2023 Jul 25;12:e83659. doi: 10.7554/eLife.83659 (PMC10368424; doi:10.7554/eLife.83659)
Supplement: Supplementary file 1. — (a) Model parameters for the base model. (b) Patient-specific parameters for the in vivo model. (c) Clinical trial information in our simulation. [file elife-83659-supp1.docx]

**Supplementary file 1** Tables for important model parameters.


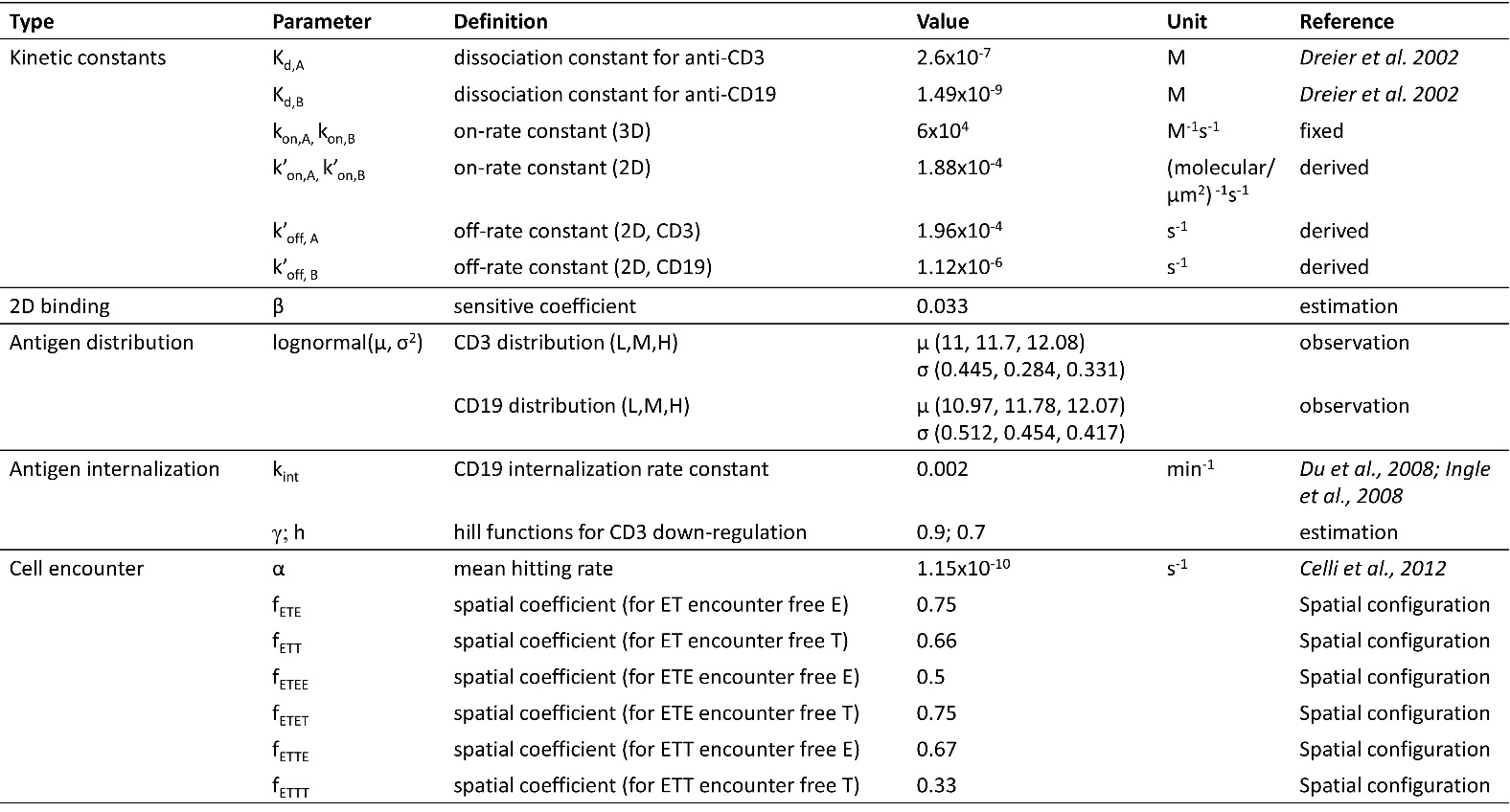
**a.** Important model parameters (for base model)


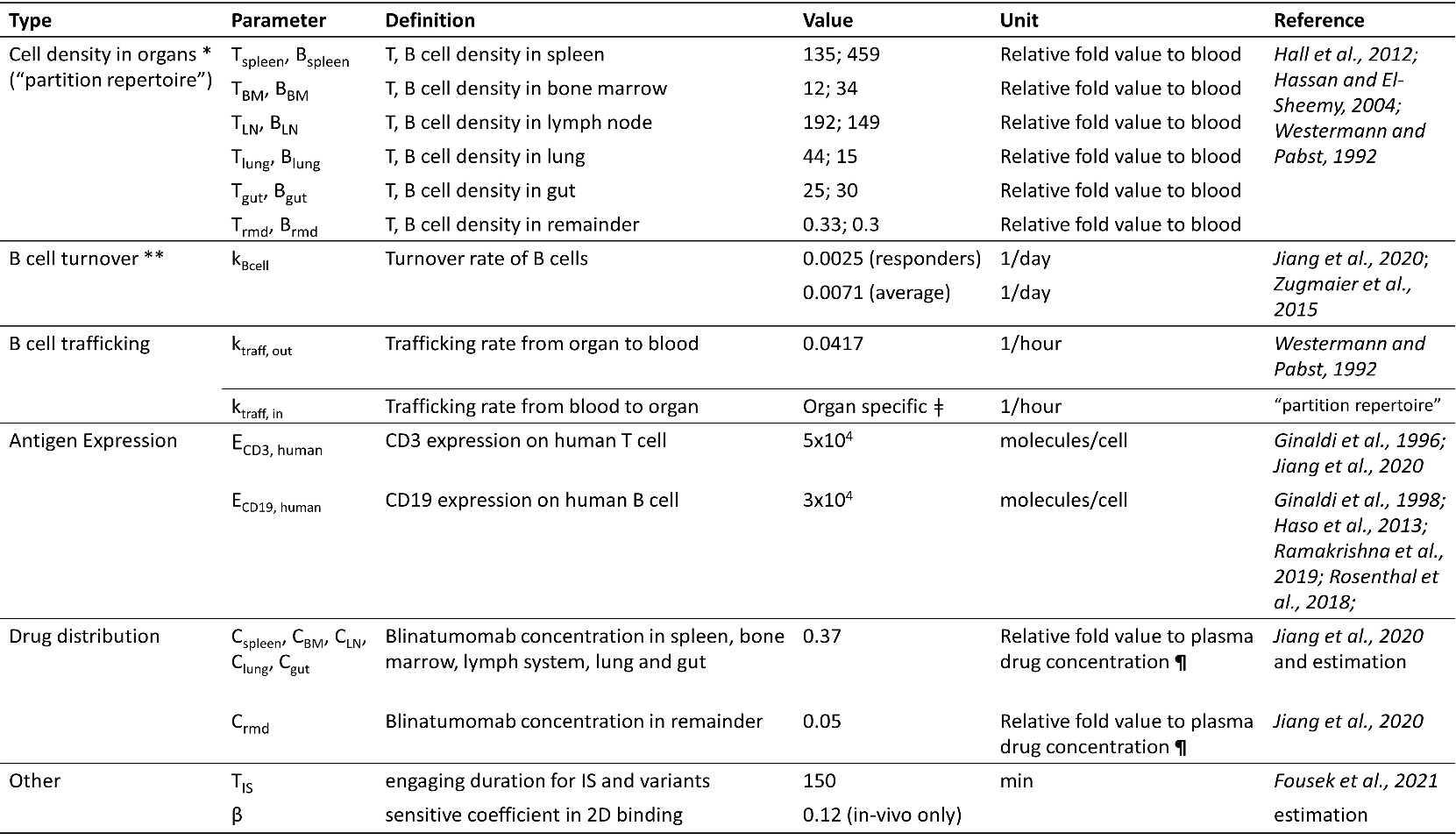
**b.** Patient specific parameters for the in-vivo model

* Patient-specific T and B cell densities at the organ level were derived based on the blood T and B cell numbers and the pre-defined “partition repertoire” (the relative fold values to blood).

** In the simulation, the value of 0.0025/day was applied to “Responder” group in trial MT103-211 and “OS>30 month” and “OS<30 month” groups in trial MT103-206; the value of 0.0071/day was applied to MT103-202, and “Non-responder” group in trial MT103-206. B cell turnover rates for trial MT103-104 and “Non-responder” group in trial MT03-211 were estimated as 0.0067/day and 0.1/day respectively.

**ǂ** k_traff, in_ for each organ was derived from “partition repertoire”.

**¶** plasma drug concentration is trial-specific, see ***Supplementary file 1c***.

Note: T cell proliferation and death rate was not included in the model. However, T cell density change was considered as an independent variable in our model. T cell density in organ during treatment was allowed to change over time in the simulation (***Figure 6b-e***). The values were derived from the change of blood T cell count based on reported data (***Bargou et al. 2008; Zhu et al., 2016; Zhu et al., 2018;*** [***Zugmaier***](https://pubmed.ncbi.nlm.nih.gov/?term=Zugmaier+G&cauthor_id=26480933) ***et al., 2015***). The blood T cell decline due to rapid redistribution after administration was excluded. However, T cell density was not allowed to change in the proof-of-concept simulations in Figure 7.


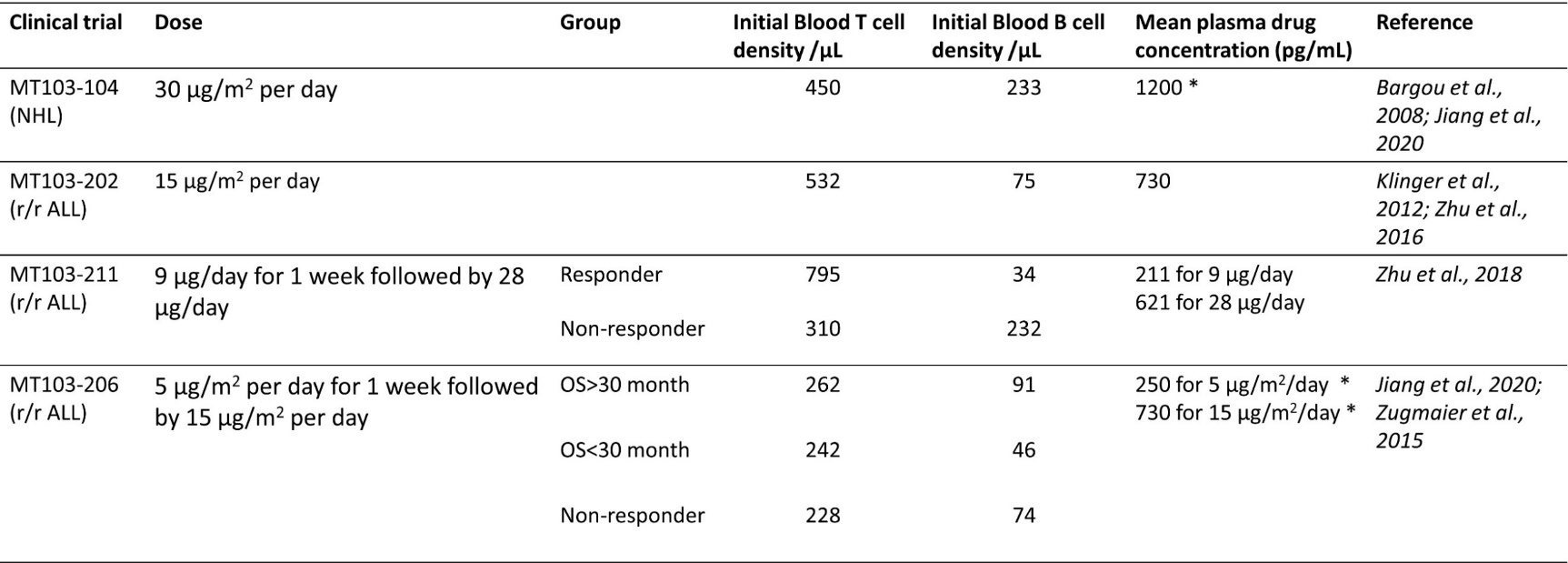
 **c.** Clinical trial information (baseline cell density in blood and mean plasma drug concentration at steady state)

NHL, non-Hodgkin's Lymphoma; OS, overall survival; r/r ALL, relapsed/refractory acute lymphoblastic leukemia.

* For MT103-104 and MT103-206, mean plasma drug concentration was derived from a linear relationship between dose and blood drug concentration (***Jiang et al., 2020***).
